# Supplementary material for: Diverse biological processes coordinate the transcriptional response to nutritional changes in a Drosophila melanogaster multiparent population
Source: BMC Genomics. 2020 Jan 28;21:84. doi: 10.1186/s12864-020-6467-6 (PMC6988245; doi:10.1186/s12864-020-6467-6)
Supplement: Supplementary file 1 — Additional file 1: Figure S1. Module detection by hierarchical clustering of 12,614 genes showing co-expressed sets of gene clusters. Figure S2. Global patterns of gene expression in 54 RNA samples. Figure S3. Histograms of the correlation between fold changes for each pair of diets, calculated with each diet as the reference diet obtained from permuted data. Figure S4. Histograms of the proportion of genes that trend in the same direction, calculated with each diet as the reference diet obtained from permuted data. Figure S5. Gene set enrichment analysis (GSEA). [file 12864_2020_6467_MOESM1_ESM.docx]

Supplementary figures

**Figure S1:** Module detection by hierarchical clustering of 12,614 genes showing co-expressed sets of gene clusters. *Top*: Selection of soft-threshold power – effect of soft-thresholding on the scale-free model fit. Because metabolic networks are likely to be scale-free, higher values of soft threshold power reduces noise of the correlations in the adjacency matrix. An optimal threshold value is one that maximizes network similarity to a scale-free graph - the lowest power for which the scale free topology index reaches 0.90 (i.e. 23 in this study, *top left*). *Top right* shows the effect of soft-threshold power on the mean connectivity. *Bottom*: Hierarchical clustering of genes using dissimilarity based on topological overlap. Height (*y*-axis*)* represents distance determined by the extent of topological overlap. A dendrogram depicts gene clusters (modules) detected. Each module is assigned a color name. Top color panel displays modules automatically detected with flashClust(). The lower panel depicts modules after merging highly correlated modules (*r* ⪰ 0.9).

**Figure S2:**

Global patterns of gene expression in 54 RNA samples. **a.** Transcript abundance as log_2_(FPKM + 1) for each sample on the x-axis: bodies, B1 - B6; heads, H1 - H6; and ovaries, O1 - O6), faceted by diet: C, DR, and HS. **b.** Distribution of transcripts per gene - the number of transcripts associated with each gene symbol. **c.** Distribution of transcript sizes - based on transcript models of annotated genes. **d.** Principal components analysis (PCA) to visualize the overall effect of diet and tissue before correcting for batch effects (compare with Figure 2 in the main text after correction for batch effect). Two dimensions are shown (PC1 and PC2) accounting for 94% of the variance in sample gene expression. FPKM - Fragments Per Kilobase Million.

**Figure S3**: Histograms of the correlation between fold changes for each pair of diets, calculated with each diet as the reference diet obtained from permuted data. The observed value is for each is shown as a vertical red line.

**Figure S4**: Histograms of the proportion of genes that trend in the same direction, calculated with each diet as the reference diet obtained from permuted data. The observed value is for each is shown as a vertical red line.

N

GO terms represent a diverse set of biological processes affected

Known nutrient sensors absent among pathways significantly enriched

**Figure S5:** Gene set enrichment analysis (GSEA). Four pathways (starting with “dme”) and 41 gene ontology terms (GO) were identified from GSEA of the whole list significantly differentially expressed genes (2,475) for the main effect of diet. GSEA differs from traditional GO analysis in that genes are weighted by their fold change in the analysis of enrichment against pre-computed gene sets. This analysis identifies four pathways that are not any of the canonical pathways for the response to diet in model organisms such as IIS/TOR pathway. Secondly, a large number of GO terms represent many other biological processes (BP) in addition to nutrient metabolism. Clearly, in this population, the response to diet is not limited to canonical nutrient sensing pathways.
