# Supplementary material for: Diverse biological processes coordinate the transcriptional response to nutritional changes in a Drosophila melanogaster multiparent population
Source: BMC Genomics. 2020 Jan 28;21:84. doi: 10.1186/s12864-020-6467-6 (PMC6988245; doi:10.1186/s12864-020-6467-6)
Supplement: Supplementary file 4 — Additional file 4. Correlation of pairwise fold changes relative to the third diet as reference. [file 12864_2020_6467_MOESM4_ESM.docx]

|  | DR v HS relative to C | | C v HS relative to DR | | C v DR relative to HS | |
| --- | --- | --- | --- | --- | --- | --- |
|  | correlation^1^ | proportion^2^ | correlation^1^ | proportion^2^ | correlation^1^ | proportion^2^ |
| Body | 0.64 (0.08) | 0.70 (0.39) | 0.33 (0.95) | 0.68 (0.44) | 0.51 (0.48) | 0.61(0.67) |
| Head | 0.59 (0.30) | 0.82 (0.01) | 0.47 (0.49) | 0.51 (0.97) | 0.43 (0.78) | 0.64 (0.65) |
| Ovary | 0.59 (0.04) | 0.66 (0.30) | 0.43 (0.95) | 0.69 (0.17) | 0.48 (0.69) | 0.49 (0.91) |

^1^ The correlation between fold changes in each diet pair. Empirical p-value is given in parentheses.

^2^ The proportion of genes that trend in the same direction for each pair of diets: number upregulated in both + number downregulated in both/total number of genes. Empirical p-value is given in parentheses.
