# Supplementary material for: Diverse biological processes coordinate the transcriptional response to nutritional changes in a Drosophila melanogaster multiparent population
Source: BMC Genomics. 2020 Jan 28;21:84. doi: 10.1186/s12864-020-6467-6 (PMC6988245; doi:10.1186/s12864-020-6467-6)
Supplement: Supplementary file 10 — Additional file 10. Composition of the four diets used in this experiment. [file 12864_2020_6467_MOESM10_ESM.docx]

Table S8: Composition of the four diets used in this experiment.

|  | **Maintenance** | **DR** | **C** | **HS** |
| --- | --- | --- | --- | --- |
| **Water (ml)** | 1066 | 1000 | 1000 | 1000 |
| **Agar (g)** | 6.25 | 10 | 10 | 10 |
| **Dextrose (g)** | 86.26 | - | - | - |
| **Sucrose (g)** | - | 50 | 50 | 342 |
| ***Molarity*** | *-* | 0.15 | 0.15 | 1 |
| **Yeast (g)** | 21.6 | 100 | 200 | 200 |
| **Cornmeal (g)** | 40.8 | - | - | - |
| **Tegosept (g)** | 1.8 | 2.7 | 2.7 | 2.7 |
| **Ethanol (ml)** | 7.3 | 11 | 11 | 11 |
| **% Protein** | 10-13 | 36-41 | 45-53 | 17-19 |
| **% Carbohydrate** | 93-95 | 59-64 | 47-52 | 81-83 |
